# Stress appraisal in the workplace and its associations with productivity and mood: Insights from a multimodal machine learning analysis

**DOI:** 10.1371/journal.pone.0296468

**Published:** 2024-01-02

**Authors:** Mohamad Awada, Burcin Becerik Gerber, Gale M. Lucas, Shawn C. Roll

**Affiliations:** 1 Sonny Astani Department of Civil and Environmental Engineering, Viterbi School of Engineering, University of Southern California, Los Angeles, California, United States of America; 2 USC Institute for Creative Technologies, University of Southern California, Los Angeles, California, United States of America; 3 Chan Division of Occupational Science and Occupational Therapy, University of Southern California, Los Angeles, California, United States of America; COMSATS University Islamabad, PAKISTAN

## Abstract

Previous studies have primarily focused on predicting stress arousal, encompassing physiological, behavioral, and psychological responses to stressors, while neglecting the examination of stress appraisal. Stress appraisal involves the cognitive evaluation of a situation as stressful or non-stressful, and as a threat/pressure or a challenge/opportunity. In this study, we investigated several research questions related to the association between states of stress appraisal (i.e., boredom, eustress, coexisting eustress-distress, distress) and various factors such as stress levels, mood, productivity, physiological and behavioral responses, as well as the most effective ML algorithms and data signals for predicting stress appraisal. The results support the Yerkes-Dodson law, showing that a moderate stress level is associated with increased productivity and positive mood, while low and high levels of stress are related to decreased productivity and negative mood, with distress overpowering eustress when they coexist. Changes in stress appraisal relative to physiological and behavioral features were examined through the lenses of stress arousal, activity engagement, and performance. An XGBOOST model achieved the best prediction accuracies of stress appraisal, reaching 82.78% when combining physiological and behavioral features and 79.55% using only the physiological dataset. The small accuracy difference of 3% indicates that physiological data alone may be adequate to accurately predict stress appraisal, and the feature importance results identified electrodermal activity, skin temperature, and blood volume pulse as the most useful physiologic features. Implementing these models within work environments can serve as a foundation for designing workplace policies, practices, and stress management strategies that prioritize the promotion of eustress while reducing distress and boredom. Such efforts can foster a supportive work environment to enhance employee well-being and productivity.

## Introduction

Work stress is a widespread problem affecting many employees. The World Health Organization has recognized work stress as a global health concern due to its implications on overall well-being and productivity [1]. According to the American Institute of Stress, 80% of workers experience job-related stress, with almost half seeking help to manage it [2]. Prolonged exposure to such stress, as noted by Ohman et al. [3], can hamper cognitive abilities, leading to compromised decision-making and problem-solving capabilities. Particularly in the realm of office work, which possesses the largest workforce in the U.S. [4], the factors contributing to work stress are manifold: long hours, pressing deadlines, overwhelming workloads, limited control over tasks, and interpersonal conflicts [5]. This stress manifests in various physical and psychological symptoms among office workers, ranging from fatigue and headaches to more severe conditions like anxiety, depression, and potential early stages of heart disease, as highlighted by Low et al. [6].

It is essential to understand that not all forms of stress experienced in the workplace are negative. Stress in the work environment can be categorized into two main types: eustress and distress. Eustress and distress are two types of stress that can occur in the workplace [7]. Eustress, or "good stress," comes from a challenging or exciting situation, such as a big project or presentation. Distress, on the other hand, occurs in overwhelming or negative situations, such as a toxic work environment or heavy workload. Eustress is linked to increased productivity and positive mood, while distress is associated with decreased productivity and negative mood [7]. Two concepts are important for differentiating eustress and distress: stress arousal and stress appraisal. Stress arousal refers to the body’s physiological, behavioral, and psychological responses to stress [8]. Stress appraisal refers to the cognitive evaluation of a situation as stressful versus non-stressful or as a threat/pressure versus a challenge/opportunity [9]. Both eustress and distress can cause stress arousal, but eustress is associated with a positive stress appraisal, where individuals see a situation as challenging but manageable, while distress is associated with a negative stress appraisal, where individuals view a situation as overwhelming and uncontrollable. It is important to note that eustress and distress can coexist in the workplace, for example, when an employee experiences eustress from a challenging project but also experiences distress due to a lack of support from colleagues [10]. This dual experience underscores the need for a balanced approach in the workplace, where both positive and negative reactions to work scenarios are considered. Relying on a holistic model for determining stress appraisal can help organizations achieve this balance, promoting positive well-being while also addressing factors related to ill-health [11].

**Fig 1:**
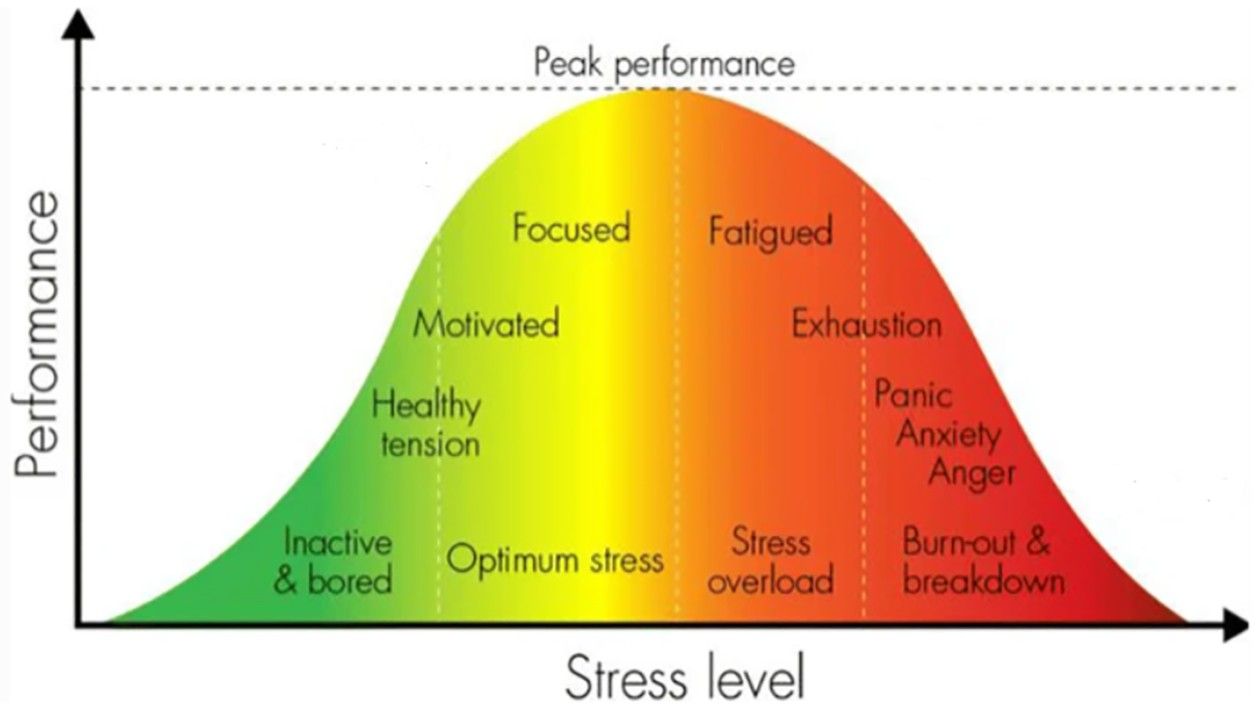
Adaptation of the Yerkes-Dodson law "inverted U-curve" for stress arousal and performance that incorporates appraisals of the experience.

The Yerkes-Dodson Law, conceptualized by psychologists Robert Yerkes and John Dodson in 1908, elucidates the intricate relationship between arousal and performance [12]. It posits that optimum performance is achieved at moderate arousal levels, whereas both low and high arousal can impede performance. Jarinto’s study [13] further delves into this, suggesting that eustress, within this framework, represents the ideal stressor level, enabling peak performance. It’s corroborated by other research which indicates that moderate pressure is conducive to optimal performance, whereas both excessive and insufficient pressure can be counterproductive [14, 15], while performance tends to deteriorate when employees encounter excessive or insufficient pressure. This relationship is elegantly depicted in Fig 1, adapted from [16]. The graph presents a spectrum: from under-challenged conditions leading to boredom, to the optimal "flow" state characterized by heightened productivity, and finally to overwhelming pressure resulting in feelings of inundation and sometimes even panic. The graph presents the inverted u-curve representing this arousal-performance relationship overlayed with potential appraisals of the experience that range from boredom to burnout. On the lower end of the graph, characterized by under-challenged conditions, individuals exhibit a lack of motivation and may approach their tasks in an unmotivated manner. In the middle section, individuals operate at their utmost efficiency, entering a state of "flow" characterized by a sense of enjoyment and heightened productivity. Conversely, the upper end of the graph depicts the struggle experienced under overwhelming pressure and bad mood, marked by the presence of numerous demands that engender feelings of being inundated and, at times, evoking panic-like responses.

A recent study targeting the prediction of office worker productivity emphasized the significance of incorporating psychological states into such models to enhance their predictive accuracy. This research specifically spotlighted mood and eustress as pivotal indicators of productivity [17]. Recognizing the impact of these four stress appraisal conditions—boredom, eustress, the coexistence of eustress and distress, and pure distress—on mood and performance enables employers to craft strategies that foster positive work environments. By mitigating extremes like burnout or boredom, these strategies can enhance both productivity and mental well-being among workers. Building on this, our study seeks to deepen the understanding of stress appraisal within the context of the Yerkes-Dodson law, conducting a quantitative examination of shifts in productivity, mood, and stress across the four stress appraisal states.

Recent research has underscored the importance of distinguishing between different types of stress when evaluating its effects on cognitive and health outcomes. One study emphasized the subdivision of stress into eustress (low-arousal) and distress (high-arousal) to better understand individual cognitive abilities and mental/physical health [18]. In this study, participants’ brain hemodynamics were assessed using functional near-infrared spectroscopy (fNIRS), which utilizes a near-infrared biosensor. By exposing participants to emotional stimulus using affective triggering images, they found that both eustress and distress groups showed brain activity in the right frontal cortex. However, the eustress group displayed heightened brain activity, while the distress group exhibited a more recessive brain activity pattern, irrespective of the type of stimuli (positive or negative). In another study, it was determined that the psychological reactions during distress resulted in distinct skin conductance responses, differing from those observed under eustress [19]. Another study showed that boredom is associated with parasympathetic nervous system activation and reduced physiological responses [20]. These findings suggest that humans exhibit varied physiological and behavioral responses based on the stress appraisal condition, establishing a foundation for differentiating between various stress appraisal conditions and further elucidating the nuances of the Yerkes-Dodson Law. Interestingly, there is a notable gap in the literature, as no studies have directly compared whether physiological or behavioral data are more suitable for examining and studying stress appraisal.

Recent advancements in Machine Learning (ML) enable the prediction of stress appraisal by analyzing patterns in physiological and behavioral data, such as Heart Rate Variability (HRV) and facial expressions. Previous studies have mainly focused on understanding and predicting stress arousal but not stress appraisal [8, 21]. However, it is crucial to investigate stress appraisal as it provides valuable insights into individuals’ cognitive evaluations of stressors, enabling a deeper understanding of their subjective experiences and potential coping strategies. For instance, it is well-established that boredom is associated with parasympathetic nervous system activation and reduced physiological responses [20], and stress arousal, whether it is positive (eustress) or negative (distress), results in heightened physiological and behavioral responses due to activation of the sympathetic nervous system [22]. Koldijk et al. [23] used this understanding to achieve 90% accuracy in detecting stress arousal using heart rate, electrodermal activity (EDA), facial features, and participants’ interactions with their computers in a controlled experimental study that mimicked office work stressors (e.g., tight deadlines, interruptions). Although important for demonstrating the utility of ML, this study did not consider individual perceptions and appraisals of stress that could moderate the relationship between arousal and worker or work outcomes. Recently, Li et al. [24] demonstrated the potential of using ML for stress appraisal prediction by achieving 70% accuracy in predicting eustress. In another study, it was discovered that a combination of facial features and physiological data (e.g., skin temperature, heart rate, blood volume pulse, and skin conductance) can accurately predict the presence of eustress with an accuracy of 83.38%, and the presence of distress with an accuracy of 78.79% [25]. However, no study has yet explored the four stress appraisal states: boredom, eustress, eustress-distress coexistence, and distress within a single machine learning framework.

Grounded on this background, the primary objective of this study is to assess the precision of ML in predicting various stress appraisal states, such as boredom, eustress, eustress-distress coexistence, and distress. Additionally, this research investigated the relationship between stress appraisal, mood, and productivity. Four research questions guided this work: (1) How do stress level (i.e., arousal), mood, and productivity differ across stress appraisal states (i.e., boredom, eustress, eustress-distress coexistence, and distress)? (2) What ML algorithms are best suited for predicting stress appraisal? (3) What data modalities are best suited for predicting stress appraisal? (4) How do physiological and behavioral responses vary across different stress appraisal states?

The structure of this paper is as follows. The Methodology section elaborates on the experimental setup for gathering data, the methods used for cleaning and processing the data, and how the various ML algorithms were trained and evaluated. The Results and Discussion section summarizes the findings, presents a discussion that provides an assessment of the potential of using ML to distinguish the four-stress appraisal states and examines the appraisal states’ associations with mood, productivity, and physiological and behavioral responses. The Conclusions section presents the conclusions drawn from the results, highlights the limitations of the study and provides suggestions for future research.

## Methodology

We conducted a controlled experimental procedure to collect physiological and behavioral data for stress appraisal prediction. This study was conducted according to the guidelines of the Declaration of Helsinki and approved by the Institutional Review Board of the University of Southern California (UP-21-00484, Effective Approval Date: 22 July 2021). All participants provided written informed consent. Data collection occurred between March 11, 2022, and July 5, 2022.

### Participants

A total of 48 healthy individuals (20 males and 28 females), primarily undergraduate and graduate students, volunteered for the experiment. Their mean age was 22.6 years (±2.1 years), and there were no dropouts; all 48 initially enrolled participants completed the study. Participants underwent a rigorous screening process using a self-report questionnaire. It is important to note that one prospective participant, who did not meet our inclusion criteria, was excluded before the study’s commencement. This exclusion was based on predefined criteria, such as vision impairments hindering computer use, psychological sensitivity to stress-inducing activities, pregnancy, or the use of medication affecting physiological signals. With this exception, all other participants successfully completed the study without any dropouts.

### Physiological, behavioral, and human-computer interaction data

During the experiment, participants were equipped with two distinct physiological monitoring devices, chosen to align with our research objectives and data collection requirements. Firstly, participants wore an Empatica E4 wristband [26], which was selected for its versatility in capturing multiple physiological parameters. The wristband collected Electrodermal Activity (EDA), Skin Temperature (ST), Blood Volume Pulse (BVP), and x, y, and z wrist acceleration. These parameters were chosen for their relevance to our study, as EDA and ST provide insights into emotional arousal and stress levels, while BVP offers information about cardiovascular responses. Additionally, the wrist acceleration data allowed us to monitor fine-grained motion-related metrics. Secondly, heart rate data was collected using an H10 Polar chest strap [27]. We chose this device due to its precision in measuring heart rate, a critical physiological metric for our study, as it reflects stress levels, emotional states, and physical exertion. In addition to physiological data, we employed behavioral data collection methods. A Microsoft Azure Kinect DK camera [28], strategically positioned atop the computer screen and facing the participant, recorded facial expressions throughout the experiment. This video data was instrumental in complementing the physiological measurements and facilitating the analysis of emotional responses. Furthermore, to gain insights into participants’ interactions with the computer, we ran the Mini Mouse Macro logging application [29] in the background. This application meticulously recorded participants’ activities involving the computer’s mouse and keyboard.

### Experimental protocol

The experiment took place in a quiet private office using a standard desktop computer. The experiment simulated 2 different work conditions: low-stress work and high-stress work.

Low-stress work: In this condition, participants had 40 minutes to prepare a PowerPoint presentation about their favorite movie, book, or television series, that is, a familiar topic. In this condition, participants worked without being monitored.

High-stress work: In this condition, participants had only 30 minutes to prepare a PowerPoint presentation about an unfamiliar topic. Participants had to present the scientific and philosophical achievements of two ancient Greek philosophers and provide their opinions about how these achievements are still shaping modern human life. The requirements (time and topic) were carefully established to make the completion of the presentation achievable but at the same time to create a sense of time pressure, heavy workload, and unfamiliarity with the task. Furthermore, a confederate played the role of a university professor who monitored the participants using live video, audio, and screen sharing via Zoom video conferencing. An application on the computer screen showed the professor’s rating of their work, which began at 100 points and then decreased and increased in a standardized manner across all participants. Changes in the score appeared at uneven intervals such that the participants could not recognize a pattern, instead, associating the scoring with the professor noticing a flaw or correction. Participants were informed that the highest-scoring individuals would receive the maximum compensation (i.e., $50) and the lowest-scoring individuals would receive minimal compensation for their time (i.e., $5). At the conclusion of the study, all participants were informed that their score did not equate to the level of compensation, and everyone received the maximum compensation (i.e., $50).

Throughout both conditions, a pop-out questionnaire appeared on the computer screen every 5 minutes, asking the participants to rate their perceived stress level, mood, and productivity. These metrics were rated using a 0–100 slider, with 0 indicating “I am not stressed at all,” “I am in a bad mood,” or “I feel extremely unproductive” and 100 indicating “I am extremely stressed,” “I am in a good mood” or “I feel extremely productive”. In addition, participants appraised their stress as distress (pressure) and eustress (opportunity/challenge) using the Valencia Eustress-Distress Appraisal Scale (VEDAS), an efficient and validated tool for appraising stress as perceived levels of distress and eustress [30, 31]. Distress was assessed using a 6-point scale as 1 (very definitely is not a source of pressure), 2 (definitely is not a source of pressure), 3 (generally is not a source of pressure), 4 (generally is a source of pressure), 5 (definitely is a source of pressure), or 6 (very definitely is a source of pressure). Eustress was assessed using a similar 6-point scale going from 1 (very definitely is not a source of opportunity/challenge) to 6 (very definitely is a source of opportunity/challenge).

Each condition started with a 5-minute baseline phase, in which participants remained idle and relaxed while we collected a baseline for all physiological signals (which is typical for stress detection research). At the end of the baseline phase, participants rated their perceived stress level and mood. Participants were given the option of taking a maximum 10-minute break if needed between the low and high stress phases; however, all 48 participants opted to proceed immediately with the next phase of the experiment. The total duration of the experiment was around 100 minutes.

### Feature extraction and data processing

The collected data was segmented into 30-second time frames to extract physiological and behavioral features. This particular time window was chosen based on the findings of Bernardes et al. [32], who determined that a 30-second duration is the smallest timeframe suitable for obtaining dependable HRV features that accurately evaluate psychological stress. Therefore, our dataset consisted of 48 participants, each with 70 minutes of data collection divided into 30-second time windows, resulting in 6720 datapoints. The final dataset comprised 83 features, including 34 physiological features, 48 behavioral features (including 3 related to human-computer interactions), 39 facial-related features, 6 features for wrist acceleration, and 1 feature indicating the participant’s gender. Table 1 provides a summary of all the features analyzed.

**Table 1:** Features dataset.

Type (Number of features)	Signal	Features Included
Physiological (34)	Electrodermal activity (EDA) Blood volume pulse (BVP) Skin temperature (ST)	Mean, Standard deviation, Median, Minimum, Maximum, 25^th^ & 75^th^ percentile, slope fitted through the data.
	Heart rate Heart rate variability (HRV)	Mean, Standard deviation, Minimum, Maximum, rmsdd, LF peak, HF peak, LF power, HF power, LF/HF
Behavioral (48)	Facial action units (AUs) Head rotation Eye gaze direction	Mean, Standard deviation
	Blink	Count
	Wrist acceleration	Mean, Standard deviation
	Mouse right clicks Mouse left clicks Keyboard keystrokes	Count
Gender (1)	Female Male	Binary

Rmsdd: Root Mean Square of the Successive Differences, LF peak: Low-frequency peak, HF peak: High-frequency peak, LF power: Low frequency power, HF power: High frequency power

Kubios software [33] was utilized to process HRV data and extract multiple time and frequency-domain indices of the heart rate signal. A moderate artifact correction was employed to identify R-R intervals varying above or below 0.25 seconds from the average. This method preserved the data’s variability while addressing the presence of any artifacts. Kubios also applied a piecewise cubic spline interpolation method to generate corrupted or missing values, resulting in a cleaner and more accurate HRV signal. The RR-interval was excluded from the feature set to prevent duplication because of its inverse proportionality with heart rate, and there was a strong 94% correlation between the two features in the dataset.

Signals obtained from the Empatica E4 were processed before feature extraction to reduce noise, similar to what was done in a previous study [34]. The BVP and ST signals were filtered using winsorization [35], a statistical technique that replaces extreme values beyond the 2^nd^ and 98^th^ percentiles. We used the MATLAB Ledalab toolbox [36] to clean and process the EDA data using a Butterworth low-pass filter, Hanning smoothing with a window size of 4 adjacent datapoints, and manual artifact correction to remove any noise that might have been caused by movement or other sources of interference. After the cleaning procedure, we calculated the mean, standard deviation, median, minimum, maximum, 25^th^ and 75^th^ percentiles, and slope of BVP, EDA, and ST to ensure a thorough assessment of the various dimensions involved in stress appraisal [8].

For behavioral data in each 30-second time window, we calculated the mean and standard deviation for x, y, and z wrist accelerations obtained from the Empatica E4, and we utilized OpenFace software [37] to extract the mean and standard deviation of participants’ facial action unit (AU) intensities from the RGB video captured by the Kinect camera. AUs are predefined facial muscle movements associated with emotions and are classified as main AUs, head movement AUs, and eye movement AUs. Facial expressions are a reliable indicator of stress and are suitable for stress detection research [23]. We removed the head translation vector in the x, y, and z planes from the analysis as it depended on the participant’s height and position in the camera frame. Similarly, we excluded head rotation in the x and y planes as it was highly correlated with the gaze vector, resulting in duplicate information. A correlation analysis confirmed the close relationship between these variables, with a Pearson correlation ranging from 89% to 94%. By removing these features, we avoided redundancy in our analysis. Lastly, we aggregated keyboard strokes and mouse clicks, which are known to be influenced by cognitive and emotional states and is a relatively innovative approach to stress prediction that has exhibited encouraging results in recent studies [38, 39].

Some data was missing from the dataset due to technical problems, including keyboard and mouse files for three participants in the low-stress condition and RGB video files for two others in the high-stress condition. To address this, an XGBoost model was trained using the existing data from 43 participants to impute the missing data. Hyperparameter tuning was conducted to optimize the model’s performance, and the best values for the learning rate, maximum tree depth, and number of trees were selected. The optimized XGBoost model was then used to predict missing data points, as this method preserves the standard deviation and shape of feature distribution and avoids data loss from deleting rows with missing entries. Mean/median imputation methods were avoided as they are less accurate [40]. Additionally, robust scaling was used per participant, a data preprocessing technique to normalize features in machine learning [41]. It employs the interquartile range (IQR) instead of mean and standard deviation, making it robust against outliers. By linearly transforming the data using the 25^th^ and 75^th^ percentiles (IQR), robust scaling ensures fair comparisons and accurate modeling, especially in the presence of outliers.

All physiological data were subtracted from individual baseline levels established before the first experimental condition to increase between-participant validity. Behavioral features were not normalized against a baseline because facial activation is closely tied to the intensity of facial expressions, which does not necessarily require normalization across participants. Perceived stress and mood data values were subtracted from the baseline; thus, the range for the perceived stress and mood variables was between -100 and 100. The baseline for perceived productivity was 0 as the participants were not performing any work; thus, the range for the perceived productivity variable was between 0 and 100.

### Outcome formulation

This study aimed to examine stress appraisal and thus required the creation of a metric to describe the four states: boredom, eustress, eustress-distress coexistence, and distress. The 6-point scales for eustress and distress were condensed by grouping the lower three and top three categories into a binary variable as "Stress not appraised as eustress" or "Stress appraised as eustress" and "Stress not appraised as distress" or "Stress appraised as distress." The two ratings were combined to classify each datapoint into one of the four stress states of interest, as shown in Table 2. The resulting dataset was unbalanced, with approximately 49% indicating eustress-distress coexistence, 28% indicating boredom, 18% indicating eustress, and 5% appraised as distress.

**Table 2:** Stress appraisal formulation and data distribution across four stress appraisal states.

Eustress Appraisal	Distress Appraisal	Stress Appraisal State	Datapoints
Stress not appraised as eustress	Stress not appraised as distress	Boredom	1890
Stress appraised as eustress	Stress not appraised as distress	Eustress	1230
Stress appraised as eustress	Stress appraised as distress	Eustress-distress coexistence	3270
Stress not appraised as eustress	Stress appraised as distress	Distress	330

### Prediction assessment

The evaluation of prediction performance involved the use of accuracy and average F_1_ score. All models presented in the results were subjected to k-fold cross-validation, with a value of 10 for k. This approach ensured that no participant was used in both the training and testing sets during any iteration of the cross-validation process, thereby enhancing the reliability and generalizability of our results.

## Results and discussion

### Perceived stress, mood, and productivity levels across stress appraisal states

**Fig 2:**
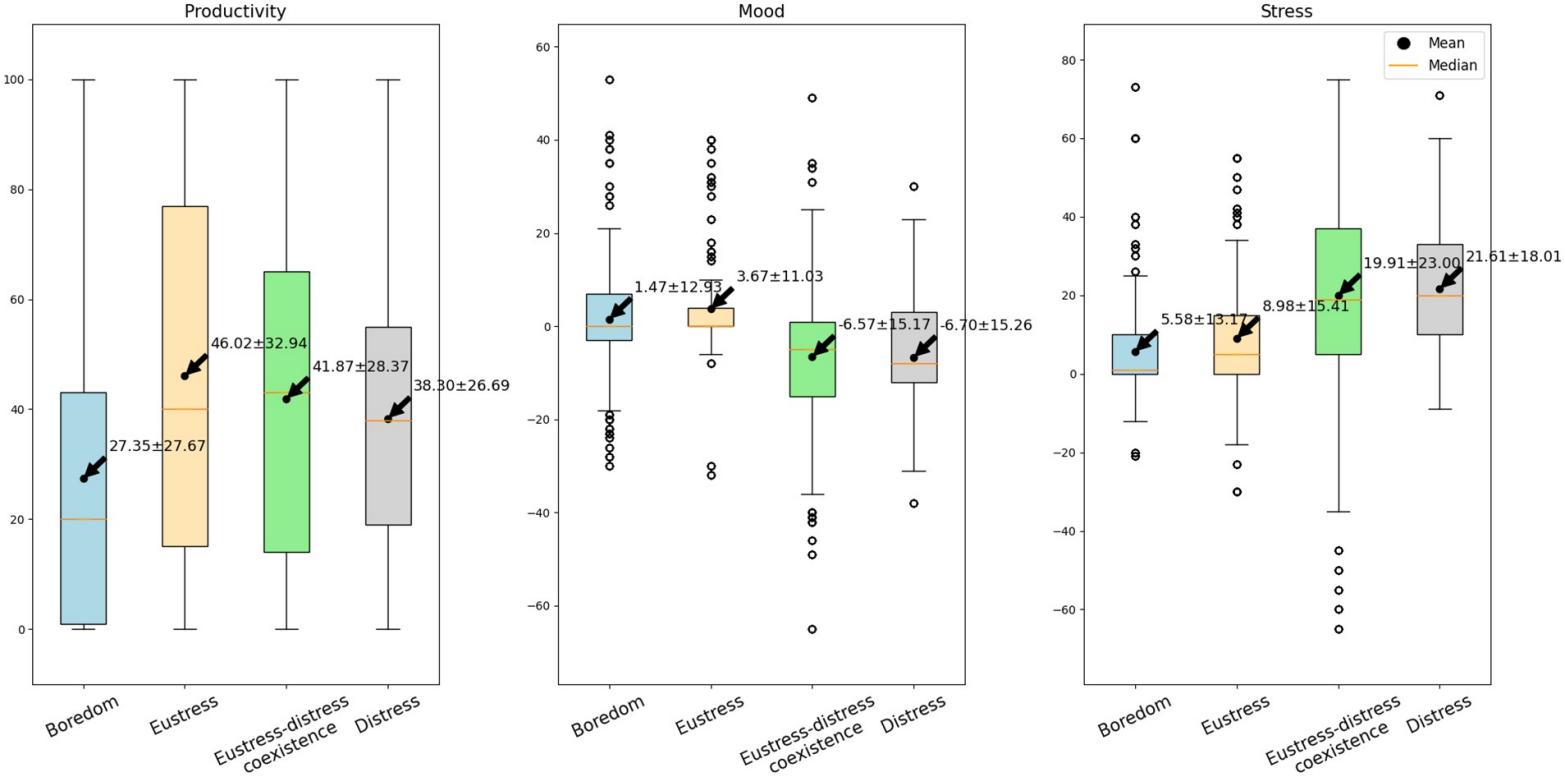
Boxplots of perceived productivity, mood, and stress across stress appraisal states.

To address the first research question of this study, we conducted three ANOVA tests to explore how different stress appraisal states relate to the perception of stress, mood, and productivity. Fig 2 presents the means and variances of these metrics across the four stress appraisal states. Statistically significant differences were identified in perceived stress (F(3, 6716) = 271.82, p<0.001), mood (F(3, 6716) = 236.62, p = <0.001), and productivity (F(3, 6716) = 135.41, p<0.001) between the four stress appraisal states. Post-hoc Tukey analysis found no significant differences in the outcomes between "eustress-distress coexistence" and "distress," but this analysis indicated all other pairwise comparisons were significant for all three metrics: productivity, mood, and stress (i.e., arousal).

The results of perceived stress and productivity generally follow the Yerkes-Dodson law. Firstly, low, or insignificant stress arousal is associated with boredom, lack of motivation, limited interest, and low performance. This is supported by our findings showing that boredom appraisals had the lowest stress level (M = 5.58±13.17) and were associated with significantly lower perceived productivity (M = 27.35±27.67) compared to the three other stress appraisal states. The Yerkes-Dodson law states that moderate stress levels can increase alertness, attention, and motivation resulting in better performance. In our results, eustress appraisals had the highest perceived productivity (M = 46.02±32.94) and a slight but statistically significant increase in stress level (M = 8.98±15.41) compared to boredom. This represents the “sweet spot” or optimal level of stress arousal that sets the grounds for eustress and maximizes performance. Next, the Yerkes-Dodson law states that as stress arousal builds up, distress takes over eustress, leading to impaired performance due to increased anxiety. This is also supported by our results, where the eustress-distress coexistence showed increased stress (M = 19.91±23.00) and reduced perceived productivity (M = 41.87±28.37). Finally, as distress became dominant, the average change in stress level was the highest (M = 21.61±18.01), and productivity showed a decreasing trend (M = 38.30±26.69).

The lack of significant difference in perceived stress and productivity between distress and the eustress-distress coexistence condition may indicate that higher stress arousal is mainly associated with distress, irrespective of whether eustress coexists with distress. Alternatively, the differentiation of these appraisal states may not have been possible due to the small number of time points appraised as distress in the dataset. Moreover, the outcomes associated with distress appraisal may be time-dependent or have a cumulative component not maximally elicited within the 40-minute experimental condition.

Changes in mood perceptions were much smaller than stress and productivity across the four appraisal states. The average rating of mood increased during times appraised as boredom and eustress, with the best mood (M = 3.67±11.03) occurring along with the eustress state. In contrast, mood was consistently rated lower than the baseline when distress was indicated, either in combination with eustress or occurring alone (M = -6.70±15.26). These results support the division of stress appraisal into 2 constructs: positive and negative. While eustress (positive construct of stress) is often associated with excitement, enthusiasm, and fulfillment [14], distress (negative construct of stress) leads to intense negative feelings [42]. It is worth noting that the negative feelings associated with distress seem to engulf the positive feelings of eustress, as demonstrated by the average perceived mood (M = -6.57±15.17) under the eustress-distress coexistence condition being almost equal to that of the distress condition.

To the best of our knowledge, this work is the first attempt to quantify the Yerkes-Dodson law using stress appraisal relative to stress arousal, performance, and mood. These findings contribute to a deeper understanding of the complex relationship between stress and human performance while highlighting the importance of optimizing stress arousal to achieve maximum productivity and well-being.

### Comparison between different ML models for stress appraisal prediction

In this section, we address the second research question of this study: What ML algorithms are best suited for predicting stress appraisal? Using all 83 features, we tested the following algorithms: Naïve Bayes, Adaboost, Logistic Regression, Linear Discriminant Analysis, Decision tree, Multilayer perceptron, Support Vector Machine (with polynomial -degree between 2 and 10- and radial kernel), K-nearest neighbor (for K-values between 2 and 15, and Euclidean or Manhattan distances), Random Forest, and XGBoost. In selecting these machine learning models, our aim was to encompass a wide spectrum of algorithmic approaches, from simple and interpretable models like Naïve Bayes and Logistic Regression to more complex and powerful methods such as neural networks (Multilayer Perceptron), ensemble techniques (AdaBoost, Random Forest, and XGBoost), and versatile kernel-based methods (Support Vector Machines and K-nearest neighbor). This comprehensive selection allows us to thoroughly evaluate the performance of various models on our dataset, ensuring that the best-suited algorithm for predicting stress appraisal is identified.

To overcome the problem of unbalanced classes, we applied an oversampling method using the synthetic minority oversampling technique (SMOTE) algorithm [43] to generate new synthetic samples in the minority classes. The algorithm draws a random sample from the minority class, identifies the k-nearest neighbors, and creates synthetic data points in the direction of the vector connecting the minority instance and its neighbors. The SMOTE algorithm was applied to the training set but not to the testing set. The results of modeling the 83 features in the training set among the ML algorithms are presented in Table 3.

**Table 3:** Comparison of ML model accuracy between different classifiers.

Algorithm	Accuracy	F_1_-score
Naïve Bayes	39.27%	40.58%
AdaBoost	48.09%	49.91%
Logistic Regression	54.94%	51.22%
Linear Discriminant Analysis	55.90%	53.61%
Decision Tree	61.07%	61.18%
Multilayer Perceptron	64.67%	63.97%
Support Vector Machine–Linear	64.55%	60.39%
Support Vector Machine–Radial	66.25%	63.57%
Support Vector Machine–Polynomial degree 5	69.06%	68.83%
K-Nearest Neighbor, K = 5, Euclidean distance	70.55%	70.42%
K-Nearest Neighbor, K = 4, Manhattan distance	75.56%	73.59%
Random Forest	77.33%	77.30%
XGBoost	82.78%	82.28%

Naïve Bayes, AdaBoost, logistic regression and linear discriminant analysis did not perform well. AdaBoost is sensitive to imbalanced datasets [44]; if one class dominates the other, it will not generalize well to new data. This is the case in our dataset, where the data distribution (Table 2) shows that the distress condition represents only 5% of the total datapoints. Naïve Bayes performs poorly with irrelevant features as its predictions may be influenced by these features that have no relation with the outcome under study. Furthermore, Naïve Bayes and logistic regression both assume that all features are independent [45], which was not true in our dataset as physiological and behavioral stress indicators are often affected by one another. For instance, sympathetic neural activity in response to stress autoregulates ST and results in EDA peaks [46]. If the input features are highly correlated, models might not be able to accurately capture the relationship between the input features and the outcome under study [44]; a correlation matrix among the input features shows that some features hold up to 80% correlation among each other. In addition, both logistic regression and linear discriminant analysis algorithms are linear models and assume that the relationship between the input variables and the output variable is linear. If the relationship is non-linear, models that can handle non-linear relationships between the input features and the output are better suited; hence decision trees, multilayer perceptron, and support vector machine algorithms led to better performance.

K-NN algorithm coupled using K = 4 and Manhattan distance showed a decent accuracy in predicting stress appraisal conditions (75.56%). K-NN is an instance-based algorithm, which makes predictions based on the similarity of new data points to the training data [47]. This means that the outcome under study is separable and can be divided into distinct classes based on the input features. However, it is worth noting that K-NN can be sensitive to the hyperparameters choice of distance metric, and the value of K as our analysis showed significant fluctuations in performance when changing these hyperparameters.

Random forest and XGBoost performed the best in predicting stress appraisal states. Random forest and XGBoost are ensemble learning methods known for their good performance on classification problems that use decision trees as the base learners, which can handle large numbers of features and are robust to overfitting. Random forest creates many decision trees and combines their predictions through majority voting, providing further robustness to overfitting and allowing for the capture of complex patterns in the data [48]. XGBoost advances this approach by using gradient boosting to optimize the decision trees and improve their performance and allows for fine-tuning of the model parameters. Additionally, XGBoost uses regularization to prevent overfitting and optimizes the tree structure to minimize the loss function. These optimizations allow XGBoost to generalize better to new data and capture more complex patterns, typically resulting in slightly better performance than random forest in classification problems [49], as demonstrated in our dataset.

### Comparison between different modalities for stress appraisal prediction

In this section, we address the third research question of this study: What data modalities are best suited for predicting stress appraisal? Our analysis first looked at clusters of data obtained from each of the primary data collection tools (e.g., E4, H10, Kinect, Mini Mouse Macro) and then explored which of the 83 individual features were most useful. Our findings here can inform which data collection methods and individual features are critical and which might be dropped to increase the feasibility of data collection in real-world settings.

First, to examine the clusters of features, we used the XGBoost algorithm for our analysis as it led to the best results, as shown in Table 3. The SMOTE algorithm was applied to all models, and gender was always included due to differences in how stress can be perceived. We trained a baseline XGBoost model based on the gender feature without any physiological or behavioral features. This model’s accuracy was 41.56%. Then, we tested several subsets of the data, as shown in Table 4.

**Table 4:** Comparison of different features and data collection tools in the prediction of stress appraisal.

Features under study* (total number of features)	Data collection tools	Accuracy	F_1_-score
Gender (1)	-	41.56%	26.13%
***1 Monitoring Device***			
EDA, ST, BVP, ACC (31)	E4	73.43%	65.67%
HR&HRV (11)	H10	63.94%	51.05%
Facial (40)	Kinect	69.46%	60.62%
Computer (4)	Mini Mouse Macro	44.87%	24.33%
***2 Monitoring Devices***			
EDA, ST, BVP, ACC, HR&HRV (41)	E4 + H10	79.55%	76.60%
EDA, ST, BVP, ACC, Facial (70)	E4 + Kinect	81.08%	78.74%
EDA, ST, BVP, ACC, Computer (34)	E4 + Mini Mouse Macro	70.42%	67.53%
HR&HRV, Facial (50)	H10 + Kinect	72.25%	65.28%
HR&HRV, Computer (14)	H10 + Mini Mouse Macro	62.17%	51.38%
Facial, Computer (43)	Kinect + Mini Mouse Macro	70.05%	60.63%
***3 Monitoring Devices***			
EDA, ST, BVP, ACC, HR&HRV, Facial (80)	E4 + H10 + Kinect	82.09%	81.56%
EDA, ST, BVP, ACC, HR&HRV, Computer (44)	E4 + H10 + Mini Mouse Macro	79.45%	76.44%
HR&HRV, Facial features, Computer (53)	H10 + Kinect + Mini Mouse Macro	75.22%	67.56%
***4 Monitoring Devices***			
EDA, ST, BVP, ACC, HR&HRV, Facial, Computer (83)	E4 + H10 + Kinect + Mini Mouse Macro	82.78%	82.28%

EDA: Electrodermal Activity; ST: Skin Temperature; BVP: Blood Volume Pulse; HR: Heart Rate; HRV: Heart Rate Variability; ACC: Acceleration

When using only 1 monitoring device, data from the E4 (accuracy = 73.43%) and facial features (accuracy = 69.46%) had the best prediction performances, while HR and HRV features resulted in weaker performance (accuracy = 63.94%), and the computer features only managed to improve the prediction accuracy of the baseline gender model by ≈3%. When adding a second monitoring device, combinations that included the E4 data had the best performance, with an accuracy as high as 81.08% when E4 data was coupled with the facial features. The results indicate that the EDA, ST, BVP, and wrist acceleration are among the biggest contributors to the accurate prediction of the different states of stress appraisal. Minimal improvement by 1% occurred when combining data from three devices (accuracy = 82.09%), almost equal to the accuracy for the full dataset comprised of all features (accuracy = 82.78%).

Importantly, a pure physiological dataset (E4+H10) resulted in a 79.55% accuracy, only 3% lower than the highest accuracy reached using all four devices (accuracy = 82.78%). This finding is important as it indicates that physiological data alone may be adequate to accurately predict stress appraisal. These results offer flexibility to users interested in implementing automated stress appraisal prediction at the workplace. If the goal is to maximize the prediction performance, collecting as many features as possible is necessary; however, when computation, time, and financial resources are limited, relying only on physiological features can provide useful results. Nowadays, a single wearable device can offer various physiological features. For instance, the Fitbit wristwatch, Oura rings, and WHOOP wristbands can collect many physiological signals such as heart rate, HRV, EDA, wrist acceleration, and oxygen saturation all at once [50], which makes them a feasible alternative for simple and unobtrusive data collection protocols and a reliable option for stress appraisal prediction in office settings.

**Fig 3:**
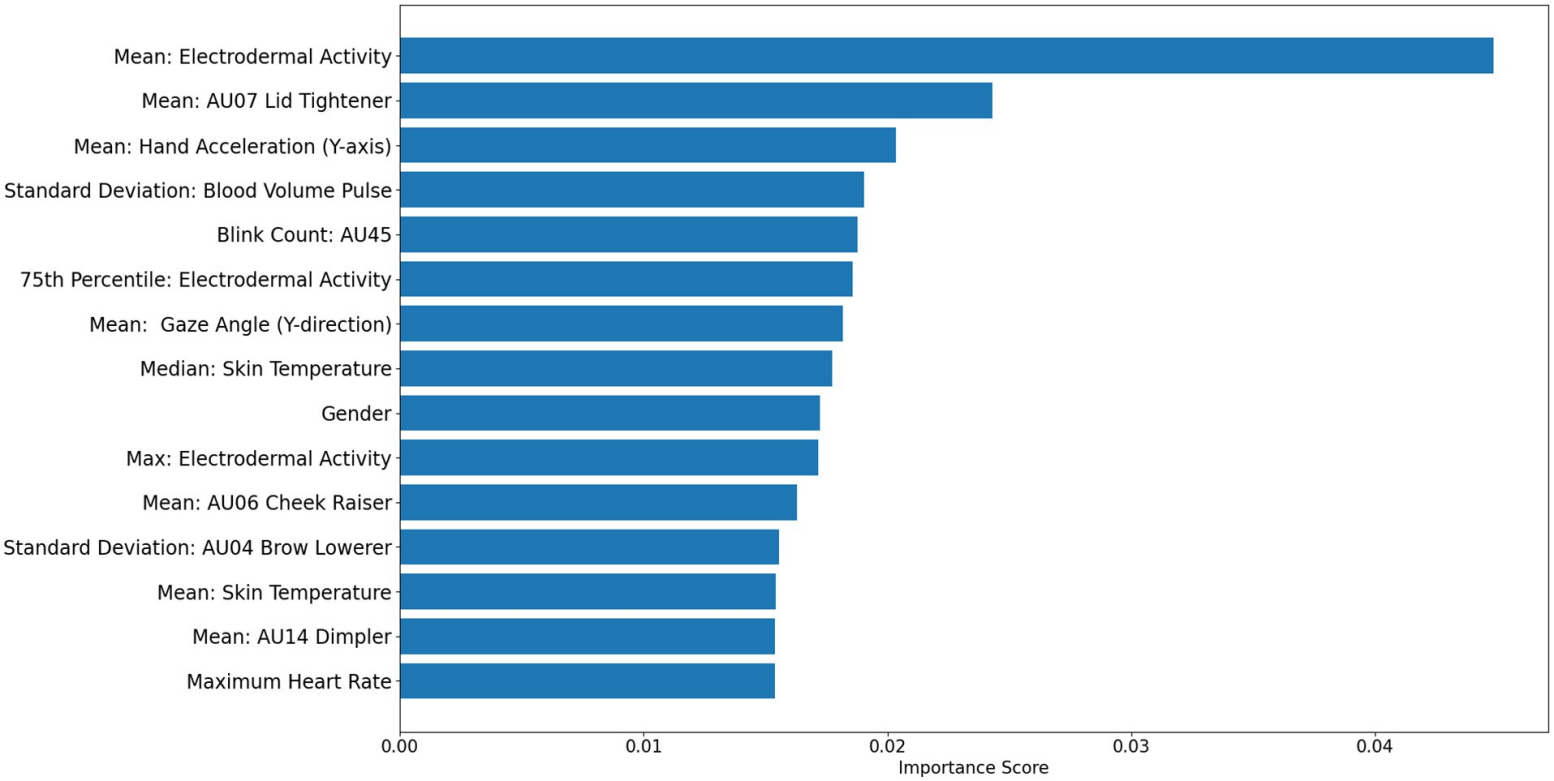
Feature importance for the stress appraisal prediction model.

To identify the individual features that had the greatest impact on predicting stress appraisal, we conducted a feature importance analysis on the best-performing ML model. We used the feature importance attribute of the model to measure how much each feature contributed to the overall prediction accuracy. An importance score for each feature was calculated to assess the contribution of each feature to the overall prediction accuracy of the ML model. Our results revealed that the 15 most important features accounted for a significant proportion of the model’s overall accuracy (accuracy _top15_ = 80.96%), leading to an almost equal prediction performance to that of the whole dataset (accuracy _whole_ = 82.78%). The 15 most important features of the stress appraisal model using combined physiological and behavioral data are presented in Fig 3.

The feature importance results indicate that EDA, ST, and BVP were the most significant physiological features for stress appraisal. Additionally, wrist acceleration (Y-axis) ranked as the third most important feature. These findings support our previous conclusions and demonstrate why the E4 wristwatch played a crucial role in improving the predictive performance of the model. The Kinect camera contributed the second most important feature (AU-07) and five other top features (AU-45, -06, -04, -14) and gaze angle (Y-direction). This highlights why a model based solely on facial features achieved a respectable prediction accuracy of 69.46% (as shown in Table 4). Heart rate and HRV features were less significant and less prevalent. The least important feature among the top 15 was maximum heart rate, which explains why a model utilizing only these features achieved an accuracy of 63.94%.

It is worth noting that gender was ranked 9^th^ in the list of important features for predicting stress appraisal states. Research has shown that gender differences affect stress arousal due to the biological nature of stress [51], and previous studies on stress detection have found that gender is an important factor that must be considered to achieve better prediction performance [39, 52]. Our results support that gender is also crucial for accurately identifying stress appraisal states using predictive models. Future studies should consider other personal factors not examined in this study that might also impact stress appraisal (e.g., age, ethnicity).

Finally, data from the Mini Mouse Macro did not contribute value to the overall prediction models, and none of the top 15 features included human-computer interaction features. While previous studies have shown that computer-related features (keyboard strokes and mouse clicks) play an important role in stress detection, our results did not find them to be a useful in predicting stress appraisal. The limited influence in our study may have been due to the controlled nature of the selected tasks that limited variability in the human-computer interactions across appraisal states. Additionally, other computer-related measures not collected in our study (e.g., keystroke pressure, pause rate, typing speed, mouse movement and wheel usage, number of open windows) might be more useful in predicting stress appraisal. Future research directions must consider additional features and examine a broader range of task conditions to provide better insights into the relationship between human-computer interaction and stress appraisal.

### Variation in physiological and behavioral signals across stress appraisal states

This section addresses the fourth research question of this study: How do physiological and behavioral responses vary across different stress appraisal states? To the best of our knowledge, this is the first study to address this research question, which makes it a unique contribution to the state of the art. We focused this analysis on the most important physiological and behavioral features for predicting stress appraisal states identified by the feature importance analysis. Table 5 summarizes the average and standard deviation values of all physiological and behavioral features based on the stress appraisal states.

**Table 5:** Physiological and behavioral data changes across the stress appraisal states.

	Boredom	Eustress	Eustress-distress coexistence	Distress
***Physiological Features***				
Electrodermal activity, mean μS*	-0.90±3.83	-0.11±0.43	0.07±0.43	0.06±0.13
Blood volume pulse, standard deviation*	38.55±27.48	46.54±26.70	51.94±35.43	50.54±22.67
Electrodermal activity, 75^th^ percentile μS*	-0.96±3.94	-0.13±0.40	0.08±0.29	0.09±0.15
Skin Temperature, median °C*	-0.48±0.77	-0.32±0.79	-0.43±0.74	-0.55±1.21
Electrodermal activity, maximum μS*	-0.82±3.80	-0.03±0.46	0.10±0.38	0.11±0.15
Skin temperature, mean °C*	-0.46±0.73	-0.42±0.76	-0.54±0.73	-0.64±1.05
Heart rate, maximum bpm*	10.74±8.21	12.96±8.58	12.32±8.29	13.27±11.25
***Behavioral Features***				
AU07 (lid tightener), mean	0.32±0.48	0.35±0.45	0.51±0.42	0.52±0.50
Wrist Acceleration in Y-axis, mean g*	4.40±17.10	10.66±14.78	9.29±14.28	5.03±15.28
AU45, blink count	7.45±4.13	7.87±4.08	6.58±4.11	6.53±4.09
Gaze angle in Y-axis, mean degrees	0.28±0.09	0.32±0.10	0.24±0.11	0.26±0.09
AU06 (cheek raiser), mean	0.15±0.28	0.19±0.25	0.17±0.26	0.04±0.12
AU04 (brow lowerer), standard deviation	0.29±0.43	0.32±0.50	0.39±0.45	0.37±0.44
AU14 (dimpler), mean	0.49±0.38	0.85±0.58	0.59±0.47	0.48±0.38

*Feature calculated as a change from the pre-experimental state with positive values indicating an increase and negative values indicating a decrease from the baseline state.

The findings indicate that EDA played a significant role in predicting stress appraisal, with three out of the top 15 features related to EDA (Table 5). Analysis of Variance (ANOVA) revealed statistically significant differences in the EDA parameters between the appraisal states: mean (F(3, 67216) = 71.09, p < 0.001), 75th percentile (F(3, 67216) = 72.98, p < 0.001), and maximum (F(3, 67216) = 74.88, p < 0.001). As in the productivity, mood, and stress analyses, post-hoc Tukey HSD tests indicated significant differences between all pairwise comparisons of EDA outcomes across the appraisal states, except for the comparisons between eustress-distress coexistence and distress conditions. All three EDA features exhibited a similar pattern. Specifically, when participants perceived their situation as boring compared to the baseline condition, there was a notable decrease in EDA with the mean, 75^th^ percentile, and maximum values of EDA of -0.90±3.83, -0.96±3.94, and -0.82±3.80, respectively. When participants perceived their situation as eustress, there was a slight reduction in EDA compared to the baseline with mean, 75^th^ percentile, and maximum values of EDA of -0.11±0.43, -0.13±0.40, and -0.03±0.46, respectively. Conversely, when participants experienced eustress-distress or distress, their EDA levels were slightly higher than baseline by 0.06–0.11 μS, an upward trend suggesting increased stress arousal [53].

Among the top 15 features used in predicting stress appraisal states, two ST-related parameters, specifically median and mean, were identified. ANOVA tests revealed a statistically significant difference in ST parameters between stress appraisal states for the median (F(3, 67216) = 14.78, p < 0.001) and mean (F(3, 67216) = 16.06, p < 0.001) values. Tukey HSD analysis demonstrated significant differences in pairwise comparisons across all four stress appraisal states. Perceptions of boredom and eustress had lower median (-0.48°C and -0.32°C, respectively) and mean (-0.46°C and -0.42°C, respectively) ST than baseline. In contrast, when participants assessed their work conditions as co-existing eustress-distress or distress alone, there was a larger decrease in both median (-0.43°C and -0.55°C, respectively) and mean (-0.54°C and -0.64°C, respectively) ST. The relationship between stress and ST is intricate and not always straightforward, which may explain why conditions characterized by low arousal (such as boredom and eustress) also exhibited a decrease in ST in comparison to the baseline. Furthermore, ST regulation involves numerous factors, including external environmental conditions, blood flow, and metabolic processes [54], which may require further exploration or control in future studies.

BVP and heart rate were the final two physiological features examined. BVP is a physiological indicator of stress arousal that measures the amount of blood pumped by the heart in one minute. When experiencing stress, the sympathetic nervous system can cause vasoconstriction, which narrows the blood vessels and decreases blood volume at the sensor placement site. This can result in lower BVP readings. Conversely, BVP readings tend to be more stable and consistent during non-stressful situations [22]. Our results support this description; the ANOVA analysis showed a significant difference in the standard deviation of BVP (F(3, 67216) = 60.28, p < 0.001) between appraisal states. More specifically, participants who appraised their work as boredom had the lowest standard deviation in BVP readings (38.55±27.48), participants who perceived their working situation as eustress showed a higher level of variation in BVP readings (46.54±26.70), and those who experienced distress had the highest level of variation (51.94±35.43–50.54±22.67). Maximum heart rate was only different in boredom states, with a significantly lower maximum heart rate (10.74±8.21) than the other appraisal states. The difference is represented by a small approximate increase of 2 beats per minute (bpm) in the maximum heart rate when comparing boredom and the other states. Maximum heart rate may be useful for studies interested in differentiating boredom, but these findings explain why maximum heart rate was the least important for predicting between all four states (Fig 3).

Among the behavioral features, our results show that wrist acceleration (F(3, 67216) = 51.50, p < 0.001) differed across stress appraisal states. wrist acceleration is the speed of movement of the hand, which can be influenced by factors such as boredom and stress. For example, boredom can result in slower hand movements, as the individual may lack motivation or interest in the task, while an adequate amount of stress can cause the hand to move more quickly, as the individual might present more engagement and be motivated and excited to finish work tasks [55]. Our results follow this pattern such that appraising the working situation as boring had the lowest wrist acceleration (4.40±17.10), and when participants appraised the work conditions as eustress, they showed the highest level of wrist acceleration (10.66±14.78). When participants appraised the work as distress, a lower wrist acceleration was noted (5.03±15.28), which aligns with an understanding that higher stress levels can interfere with motor performance and motor skills due to muscle tension and decreased dexterity, contributing to lower wrist acceleration. No statistically significant differences were noted for wrist acceleration between appraisals of the coexistence of eustress and distress (9.29±14.28) and the eustress (10.66±14.78). Just as previous studies have indicated the value of acceleration data for predicting stress arousal [56], our data indicate this feature may be useful in differentiating ideal stress appraisal states (i.e., those containing eustress) from less ideal states (i.e., boredom and distress).

Eustress, distress, and boredom can all have a significant impact on facial features and body language. When a person experiences eustress, their facial expression can reflect excitement and engagement, with increased animation in the eyes, eyebrows, and mouth. On the other hand, when a person experiences distress, their facial expression can be tense and tight, with furrowed eyebrows, a tight mouth, and a downward gaze. Boredom can result in a neutral or expressionless face, little movement in the eyes or eyebrows, and a passive or downward gaze. These emotions can also impact body language, such as head rotation or gaze direction, as people tend to lean away from negative stimuli and towards positive ones. These changes in facial features can provide valuable insights into a person’s emotional state and can be useful in fields such as psychology, human-computer interaction, and neuroscience. In general, when a person is experiencing stress arousal, (eustress or distress), the intensity of AU07 (lid tightener) tends to increase [57], while the changes in the intensity of AU04 (brow lowerer) also increases, although to a lesser extent [58]. This pattern of facial movements is often associated with a tense or anxious expression, which may reflect a heightened state of arousal in response to stress. On the other hand, when a person is experiencing boredom, the intensity of both AUs 07 and 04 tends to decrease, which is associated with a relaxed or neutral expression. This pattern of facial movements may reflect a decreased state of arousal in response to a lack of stimulation or challenge. This description is in line with the findings presented in Table 5.

Other facial features can be analyzed from the perspective of engagement, interest, and enthusiasm about work tasks, and therefore their relation to productivity and performance of individuals. High levels of engagement and productivity are associated with positive emotions such as happiness, interest, and motivation. These emotions can cause an increase in the activation of the zygomaticus major muscle (AU06: cheek raiser), which pulls the corners of the mouth upwards and creates a subtle smile [44]. Additionally, high engagement and productivity can lead to an increase in the activation of the orbicularis oculi muscle (AU14: dimpler), which raises the upper lip. This action unit has been widely used in ML applications for the detection of increased attention and activity engagement [59, 60]. Our findings align with these concepts, showing that the intensity of the action units is low when participants experience boredom or when distress arises as performance degrades. On the other hand, the AU14 intensity reaches high levels during a eustress state associated with optimal performance. It is important to note that these relationships are complex and can vary between individuals.

The ANOVA results also suggest significant differences between stress appraisal states in blink count (F(3, 67216) = 5.01, p = 0.02). The blink count of 7.45±4.13 while experiencing boredom was slightly lower than during eustress states (7.87±4.08). Although not statistically significant, this observation indicates that eustress may contribute to a heightened level of attention or cognitive engagement, leading to an increased blink rate. In contrast, participants experiencing distress, representing a negative and aversive emotional state, exhibited a lower blink count. Specifically, participants demonstrated blink rates of 6.58±4.11 and 6.53±4.09 under eustress-distress coexistence and pure distress conditions, respectively. Post-hoc tests indicated significant differences between the pairwise comparisons of eustress-distress coexistence with both boredom and eustress states. These findings suggest that distress may elicit a distinct physiological response compared to boredom and eustress. The lower blink rate during distress could indicate heightened vigilance or cognitive load, as individuals may be more focused on the distressing stimuli, resulting in a reduced blink rate.

Lastly, the mean gaze angle in the y-direction is an important feature for predicting stress appraisal. However, these features are often affected by other factors, such as bodily postures [61], which were not examined in our analysis. Further analysis is needed to determine the specific impact of this feature beyond the effects of posture to ensure a more accurate and comprehensive understanding of the relationships between stress arousal and appraisal, and also performance, and the role played by mean gaze angle in these relationships. Furthermore, while it is noteworthy that individuals can exhibit unique variations in their facial expressions, the connection between stress arousal and certain facial movements may differ from person to person. Additionally, context, personality, personal traits, and other factors can impact the correlation between performance, engagement, and activation of facial muscles. To gain a comprehensive understanding of the intricate relationship between stress arousal, eustress, distress, boredom, and performance with facial expressions and gaze, further research is needed.

## Conclusions

To the best of our knowledge, this research is the first to use a ML framework to forecast stress appraisal. The stress appraisal was separated into four categories: boredom, eustress, combined eustress-distress, and distress. The study simulated various work scenarios with two levels of stress arousal: low-stress and high-stress work conditions. To build the ML prediction models, both physiological and behavioral signals were utilized. The findings demonstrate that the relationship between perceived stress and performance aligns with the Yerkes-Dodson principle, where moderate stress is associated with improved performance but too much or too little stress is linked with degraded performance. After evaluating thirteen different classifiers, our results indicate that the XGBoost algorithm had the best performance for predicting stress appraisal states. By utilizing this model, a combination of physiological and behavioral features resulted in an accuracy rate of 82.78% in predicting stress appraisal. The feature importance analysis showed that physiological and facial features had the greatest impact on prediction performance, while human-computer interaction features had little effect. Finally, we explored how the intensities of physiological and facial features varied across the different stress appraisal conditions and analyzed these differences in relation to previous research findings in the field.

### Practical implications

Our research extends beyond the realm of academic inquiry, intertwining the fields of general psychology, organizational psychology, and affective computing to offer practical solutions for enhancing the well-being and health of employees within the workplace. Rooted in the principles of general psychology, our study delves deep into the intricacies of stress appraisal and its implications for human physiology, behavior and cognition. From an organizational psychology perspective, in the context of today’s fast-paced and demanding work environments, addressing stress-related issues is paramount to eliminating burnout, mitigating intense stress, and preventing extended stress exposure. First and foremost, our research empowers managers with a valuable tool to enhance the well-being of their teams. By utilizing our stress appraisal model, managers can gain insights into their employees’ unique stress appraisals, which encompass not only distress but also eustress–the beneficial form of stress associated with motivation and performance enhancement. Armed with this knowledge, they can make more informed decisions when it comes to task allocation. This approach goes beyond mere task distribution; it considers individual stress thresholds and preferences, creating opportunities to harness the power of eustress, prevent boredom situations, and eliminate extended periods of excessive distress which can be significant contributors to burnout.

Acknowledging the financial considerations that organizations often face, we propose a cost-effective approach to monitoring employee well-being. We recommend the utilization of a single monitoring device for data collection. Drawing from the domain of affective computing, this pragmatic approach strikes a balance between effectiveness and cost-efficiency, leveraging technology to understand and respond to human emotions and physiological responses. It is vital to emphasize that the implementation of such monitoring systems should be accompanied by transparent communication and a steadfast commitment to respecting privacy rights. When employees are informed about the purpose behind data collection and have the agency to choose their level of participation, trust is fostered, and sustainable well-being initiatives are better supported.

Employee engagement and job satisfaction are pivotal components of a healthy work environment. Our stress appraisal model offers a valuable tool for identifying instances of employee boredom, distress, and eustress. Recognizing the positive impact of eustress on motivation and performance, organizations can develop tailored strategies not only to eliminate distress but also to harness the power of eustress, thereby contributing to improved well-being. Effective stress management is essential in maintaining a healthy workplace. Beyond assessing stress arousal, our model can evaluate the effectiveness of stress-management training programs. By considering stress appraisal alongside stress arousal, organizations gain a more comprehensive understanding of how well these programs are truly benefiting employees. This insight can lead to more targeted and effective stress management initiatives, further supporting well-being.

To further enhance our framework, we propose coupling it with a notification system that can alert workers to prolonged distress experiences. This proactive approach allows for timely intervention suggestions to limit unhealthy stress exposure. By intervening early and promoting the appropriate balance between distress and eustress, organizations can prevent the escalation of stress-related issues, promoting the well-being and health of their employees.

### Limitations and future research directions

The study, which represents the first effort to use ML to identify stress appraisal states, has some limitations that should be addressed in future research. One limitation is that the experiment was not a true reflection of office work, as participants were assigned predesigned tasks and subjected to prescribed work conditions. To that end, it should be noted that the importance of different signals, such as wrist acceleration, may vary depending on the nature of the task or job description and should. Hence, future research is needed to examine stress appraisal within real-life work environments. The ML models in this study focused on gender as a moderator in stress appraisal, but they did not encompass all the personal factors affecting stress. While our study advanced stress appraisal through machine learning, it is crucial to recognize that factors beyond gender, like age, socioeconomic background, early life experiences, and ethnicity, also impact stress responses. To fully understand stress experiences, future research should explore these factors to enhance ML models and establish personalized prediction frameworks for distinct worker groups based on their unique profiles. Finally, the results showed that both head movement and gaze are important predictors of stress appraisal, suggesting the importance of considering body posture in future research.

## Supporting information

S1 Dataset (XLSX)
